# Metformin activated AMPK signaling contributes to the alleviation of LPS-induced inflammatory responses in bovine mammary epithelial cells

**DOI:** 10.1186/s12917-021-02797-x

**Published:** 2021-03-01

**Authors:** Tianle Xu, Xinyue Wu, Xubin Lu, Yusheng Liang, Yongjiang Mao, Juan J. Loor, Zhangping Yang

**Affiliations:** 1 College of Animal Science and Technology, Yangzhou University, Yangzhou, 225009 People’s Republic of China; 2 Joint International Research Laboratory of Agriculture and Agri-product Safety of Ministry of Education of China, Yangzhou University, Yangzhou, 225009 People’s Republic of China; 3 Mammalian NutriPhysioGenomics, Department of Animal Sciences and Division of Nutritional Sciences, University of Illinois at Urbana-Champaign, Urbana, 61801 USA

**Keywords:** pbMEC, Inflammation, Metformin, AMPK signaling, Metabolic changes

## Abstract

**Background:**

Lipopolysaccharides (LPS) derived from gram-negative bacterial are often regarded as primary inducer of bovine mammary inflammation. This study evaluated the biological response of metformin activated AMPK signaling on LPS-induced inflammatory responses and metabolic changes in primary bovine mammary epithelial cells (pbMEC). The pbMEC were exposed to either 3 mmol/L Metf. for 12 h as Metf. group (**Metf.**) or 2 μg/mL LPS for 6 h as LPS group (**LPS**). Cells pretreated with 3 mmol/L metformin for 12 h followed by washing and 2 μg/mL LPS exposure for 6 h were served as ML group (**ML**). PBS was added to cells as the control group (**Con.**).

**Results:**

Pre-incubation with Metf. inhibited LPS-induced expression of pro-inflammatory genes (*TNF*, *IL1B*, *IL6*, *CXCL8*, *MYD88* and *TLR4*) and proteins (IL-1β, TNF-α, NLRP3, Caspase1, ASC) and was accompanied by increased activation of AMPK signaling. Compared with the LPS group, phosphorylation of p65 and IκBα in the ML group were decreased and accumulation of NF-κB in the nucleus was significantly reduced by pretreatment with metformin. Metformin protects the cells from the increase of LPS-induced binding activity of NF-κB on both *TNFA* and *IL1B* promoters. Compared with the LPS group, genes (*G6PC*, *PCK2*) and proteins (SREBP1, SCD1) related to lipogenesis and carbohydrate metabolism were downregulated while catabolic ones (*PPARA*, *ACSL1*, *Glut1*, *HK1*) were upregulated in the ML group. Furthermore, increased acetylation of H3K14 by LPS challenge was reversed by pretreatment with metformin.

**Conclusion:**

Altogether, our results indicated that pretreatment with metformin dampens LPS-induced inflammatory responses mediated in part by AMPK/NF-κB/NLRP3 signaling and modification of histone H3K14 deacetylation and metabolic changes.

**Supplementary Information:**

The online version contains supplementary material available at 10.1186/s12917-021-02797-x.

## Introduction

Mastitis is a frequent disease of lactating dairy cows and remains a major economic threat to the dairy industry [1]. Gram-negative pathogens such as *Escherichia coli* (*E. coli*) frequently elicit clinical symptoms [2, 3]. In general, these effects include reduction of milk synthesis and a dramatic inhibition of casein synthesis mediated in part through dysregulation of protein and lipid metabolism [4, 5]. The often-generalized inflammatory response during *E. coli* mastitis is caused partly through release of lipopolysaccharide (LPS), a component of the primary cell envelop in Gram-negative bacteria. Stimuli such as LPS trigger systemic inflammation mainly through toll like receptors (TLRs) in combination with other molecules such as myeloid differentiation factor 88 (MyD88) and nuclear factor-κB (NF-κB) [6, 7]. In addition to increased secretion of inflammatory cytokines and chemokines during inflammation, the activation of NF-κB signaling also induces shifts in cellular energy metabolism through alterations in AMP-activated protein kinase (AMPK) and sirtuin 1 (SIRT1) [8, 9].

AMPK is a key regulator of numerous metabolic pathways, including lipid metabolism, glucose metabolism, and energy homeostasis [10, 11]. In bovine hepatocytes, the LPS-stimulated inflammatory response was attenuated by addition of sodium butyrate and AMPK was involved as a key regulator during the process [12]. Because sodium butyrate acts as an inhibitor of histone deacetylase, the mechanism may involve histone modifications [13]. A study in bovine retinal capillary endothelial cells reported that sirtuin 1 (SIRT1) regulated inflammation and apoptosis through an AMPK-dependent pathway [14]. Furthermore, AMPK activation inhibits cytokine-induced NF-κB activation in vascular endothelial cells [15]. Whether AMPK plays a positive role in the regulation of energy metabolism and inflammation in bovine mammary cells remains unknown.

Metformin (1, 1-dimethylbiguanide hydrochloride) was originally developed from natural compounds found in the plant *Galega officinalis*, known as French lilac or goat’s rue and has been a first-line therapy for the treatment of type 2 diabetes for decades [16]. Anti-inflammatory and anti-carcinogenic actions of metformin have been evaluated in recent studies [17–19]. Increased expression of inflammatory genes results in greater energy demands and, thus, energy expenditure pathways are switched on to support the acute-phase response [5]. However, the energy homeostasis regulator AMPK is suggested to be dependently or independently involved in the anti-inflammation effect of metformin [20, 21]. In addition to the role of AMPK activation, metformin is proposed to modulate host immune responses to infections, including the recruitment of neutrophils and mitigation of inflammatory responses [22, 23]. The negative consequences of bovine mastitis on inflammation are an area of active research. Antibiotics are the most-common substances used to cure mastitis and reduce tissue inflammation, but there is growing concern that these compounds eventually lead to resistant bacteria and lower their susceptibility to multidrug treatments [24]. In the current study, we aimed to investigate the potential effects of metformin on the LPS-induced immune response and relevant metabolic changes in primary bovine mammary epithelial cells.

## Materials and methods

### Chemicals

Metformin was purchased from Sigma (D150959, Sigma-Aldrich, St. Louis, US) with a purity of more than 97%. The lipopolysaccharide used in all experiments was from *Escherichia coli* O111:B4 lyophilized powder (L2630 from Sigma, St. Louis, MO).

### Ethics

All experimental procedures were approved by the Animal Experiment Committee of Yangzhou University, in accordance with the Regulations for the Administration of Affairs Concerning Experimental Animals (The State Science and Technology Commission of China, 1988) published by the Ministry of Science and Technology, China, in 2004. All of the experimental protocols were performed in accordance with the approved guidelines and regulations.

### Cell culture conditions

Culture of primary bovine mammary epithelial cells (**pbMEC**) was performed as described previously [25, 26]. Briefly, mammary tissue obtained from 3 lactating dairy cows without incidence of clinical disease was use for cell isolation and purification. All experiments were performed with cells at the 4 to 6 passage. Cells (2 × 10^5^) were seeded in 6-well plates with overnight incubation in complete medium (90% RPMI 1640, 8,119,417 Gibco, CA, and antibiotics (penicillin 100 IU/ml; streptomycin 100 μg/ml)). All medium supplements including 10% fetal bovine serum were purchased from Gibco (Thermo Fisher Scientific, CA). Cells were maintained at 37 °C in a humidified 5% CO_2_ incubator until reaching confluence.

### Experimental design

The inflammation model was tested and optimized with respect to LPS and metformin concentrations as well as the timing of inflammatory responses. Cells were challenged using either metformin for 12 h (metformin group, **Metf.**) or LPS administration for 6 h (LPS group, **LPS**). The control group (**Con.**) was supplied with PBS. Experiments were performed using pretreatment with metformin for 12 h, followed by washing and then LPS exposure for 6 h (ML group, **ML**). Cells were cultured with serum-free medium prior to treatment with 2 μg / mL of LPS and 3 mmol / L of metformin.

### Cell viability

Viability of pbMEC was quantified using the CCK-8 Cell Counting kit (Vazyme, Nanjing, China) according manufacturer’s protocols. Briefly, pbMEC were seeded into 96-well plates at 1 × 10^5^ cells/mL. After 24 h incubation, cells in each well with specific treatments were incubated with 10 mL of CCK-8 at 37 °C for 2 h before measuring OD at 450 nm with a microplate reader (SPARK, TECAN, Switzerland).

### ELISA for cytokine analysis

Concentrations of IL-6 and TNF-α in the cultured medium were determined by ELISA, which was conducted using the DuoSet ELISA bovine IL-6 (R&D Systems, Minneapolis, MN) and DuoSet ELISA bovine TNF-α (R&D Systems, Minneapolis, MN) kits in accordance with the manufacturer’s instructions.

### RNA extraction and quantitative real-time PCR analysis

Total RNA was isolated with RNAiso Plus* (No.: 9108) according to the manufacturer’s instructions (https://www.takarabiomed.com.cn/DownLoad/9108Q.pdf). cDNA was synthesized using Advantage RT-for-PCR Kit (Clontech) and then purified with a purification kit (Axygen, Tewksbury, MA). qRT-PCR was performed using HiScript II One Step qRT-PCR SYBR Green Kit (Vazyme) on an Applied Biosystems 7300 Real-Time PCR System (Applied Biosystems, Foster City, CA) according to a previous study [27]. Primers were designed with Premier 6.0 software (Premier Biosoft International, Palo Alto, CA) as shown in previous publications [28, 29]. The genes *GAPDH*, *RPS9*, and *UXT* were used as internal control genes. The geometric mean of the internal control genes was used to normalize target gene expression data. The validity of internal control genes as references for normalizing gene expression in mammary samples has been previously reported [30]. The 2^−ΔΔCt^ method was used for relative quantification [31].

### Western blotting

Western blot was performed using protocols described previously [25]. Briefly, equal amounts of protein isolated from pbMEC by RIPA lysis buffer (Beyotime, Shanghai, China) were separated on 10% SDS polyacrylamide gels. Proteins were transferred onto nitrocellulose membranes (Millipore, Billerica, MA), which were incubated with primary antibodies overnight at 4 °C. After washing 6 times, the blots were incubated with horseradish peroxidase-coupled secondary antibodies. Differences in protein transfer efficiency between blots were normalized with GAPDH quantification. The gray values of the bands of each target proteins were quantified with Bio-Rad image system analysis software (Bio-Rad, Hercules, CA). Primary antibodies for p-P65, p-IκBα, IκBα, NLRP3, Caspase-1, ASC, IL1β, TNFα, acetyl-H3K14, histone H3, p-AMPKα, AMPKα, p-ACCα, ACCα, GLUT1 and SCD1 were purchased from Cell Signaling Technology (Danvers, MA) (#3033, #2859, #4812, #15101, #3866, #67824, #12703, #6945, #7627, #4499, #2535, #5831, #11818, #3676, #12939, #2794), and p65 was purchased from abcam (ab16502) and were diluted 1:1000 for incubation. Primary antibodies for SREBP1c and GAPDH were purchased from Abcam Corporation (ab6728 and ab8245) and were diluted 1:5000 for incubation.

### Immunofluorescence

Immunofluorescence was performed using protocols described previously [27]. Mammary cells (2 × 10^4^ cells/well) were plated onto 12-well plates, fixed with 4% paraformaldehyde for 15 min, then washed 3 times with PBS and incubated with 0.3% or 0.5% Triton X-100 for 15 min at room temperature to increase the permeability. Cells were washed three times with PBS, incubated for 1 h with 5% BSA at 37 °C, and then incubated at 4 °C overnight with primary antibody (the same as that used in the Western blot analysis) in PBS containing 1% BSA and 0.3 Triton X-100 (T9284, Sigma-Aldrich). After PBS washes, cells were stained for 1 h with FITC labeled goat anti-rabbit FITC secondary antibody in a dark 37 °C room and then washed 3 times with PBS. DAPI (1 μg/mL) (D8417, Sigma-Aldrich) was used for nuclear counterstaining for 5 min, and then cells were washed 3 times. Cells were imaged using a DMi8 Microsystems GmbH (Leica, Wetzlar, Germany).

### Chromatin immunoprecipitation assay

Preparation of samples and experiment were performed according to the protocols described previously [29]. Briefly, cells were seeded into 6-well plates for treatment and harvested using in PBS containing protease inhibitor cocktail (Cat. #11697498001; Roche, Basel, Switzerland). Formaldehyde at concentration of 1% were added for the cross-link of protein and DNA. After 10 min shaking, glycine was used for the stop of the reaction. The mix was then centrifuge at 4 °C with 4000×g for 5 min. Chromatin preparations were fragmented at 200 to 500 bp in length with sonication on ice. Chromatin preparations were incubated with 4 μg of primary antibody (Anti-P65, ab16502, Abcam) at 4 °C for 16 h. Rabbit IgG was incubated with sample as negative control. Protein A/G agarose beads (40 μL, 50% slurry, sc-2003; Santa Cruz Biotechnology) was for capturing immunoprecipitated chromatin complexes and 200 μL of chromatin preparation served as input. Promoter fragments harvested during chromatin immunoprecipitation were quantified with qPCR using primers specific for the respective areas of the *TNFA* and *IL1B* promoter. (TNFA: forward 5′ GACAGAAGGTG TAGGGCCAG 3′ and reverse 5′ CGCTCTGGGAGCTTCTCT 3′; IL1B: forward 5′ GGCT CAGCTTGTAAAGAATC 3′ and reverse 5′ GAATGCACGAAAGTC ATCC 3′).

### Statistics

Data are expressed as the means ± standard error of the means (mean ± SEM). All data were analyzed using one-way ANOVA with Dunnett’s post-test by SAS Statistics (v 9.2, SAS Institute Inc., Cary, NC). Differences with *P*-values < 0.05 were considered statistically significant. Experiments were performed in triplicate, with three replicates in each experiment.

## Results

### Bovine mammary epithelial cells viability with LPS challenge and metformin treatment at different doses

**Fig. 1:**
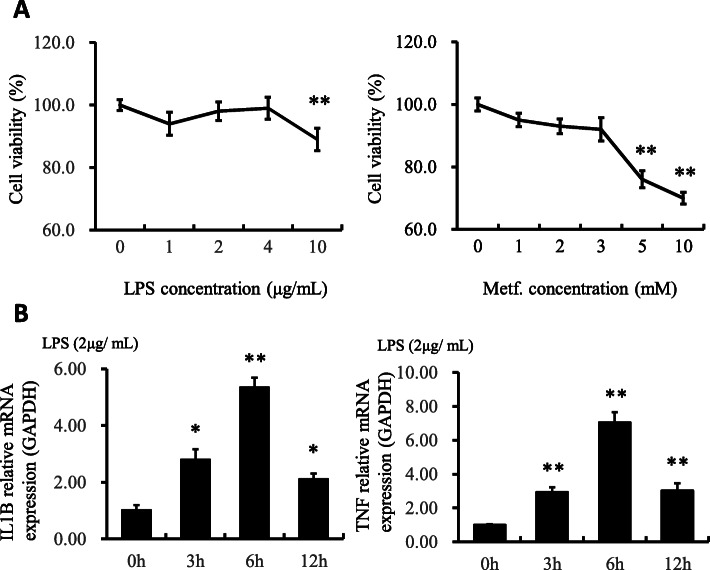
Viability of cells treated with different amounts of LPS and metformin. **a** The results are expressed as the means ± SD. **P < 0.01 vs the cells treated with 0 μg/mL of LPS and metformin. **b** Time-dependent effect. Cells were treated with LPS at 2 μg/mL for different time at 0 h, 3 h, 6 h and 12 h, respectively. The results are expressed as the means ± SD. * *P* < 0.05 and **P < 0.01 vs the cells treated with 2 μg/mL of LPS for 0 h. The application of metformin at 3 mM and LPS at 2 μg/mL for 6 h are optimal for the following experiments

Viability of pbMEC exposed to different doses of LPS and metformin was assessed via CCK-8. Cells were treated with LPS and metformin for 6 h and 12 h. As shown in Fig. 1, viability of cells challenged with LPS (1, 2, or 4 μg/mL) or metformin (1, 2, or 3 mmol/L) did not differ from controls. In contrast, compared with the respective controls, LPS at 10 μg/mL and metformin at 5 or 10 mmol/L induced lower cell viability. Thus, results suggested that to prevent negative effects on cell viability optimal dose of LPS was 2 μg/mL and metformin 3 mmol/L. Furthermore, we examined the effect of LPS at 2 μg/mL over time on expression of *IL1B* and *TNF* mRNA. Compared with the control group, both *IL1B* and *TNF* were upregulated to the highest extent at 6 h. Thus, to achieve the desired inflammatory response without affecting cell viability, we chose 6 h as the optimal time for treatment of LPS at a concentration of 2 μg/mL.

### Metformin induced activation of AMPK signaling pathway

**Fig. 2:**
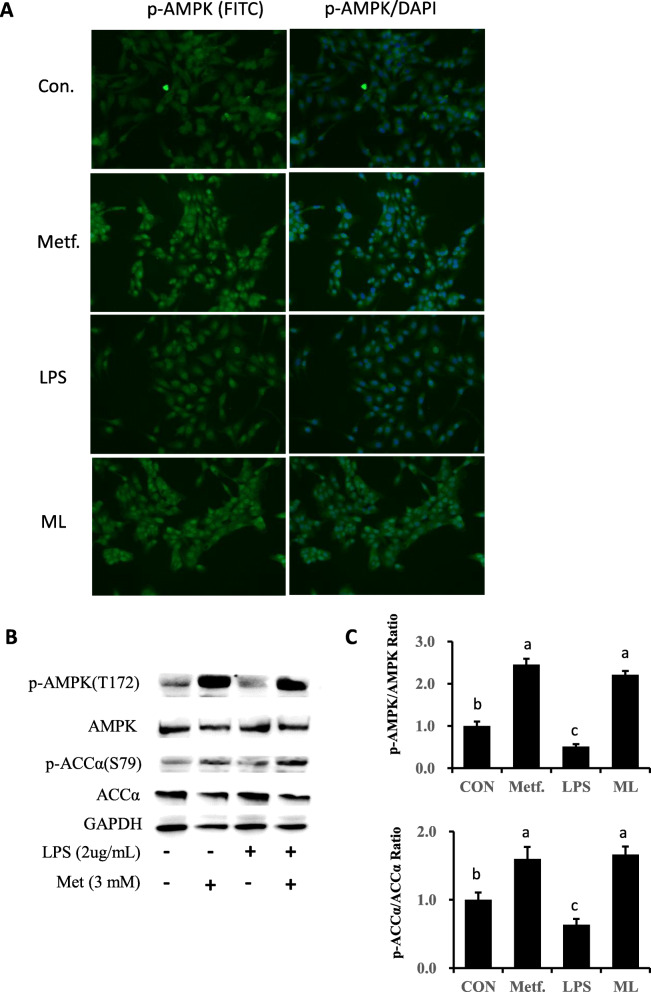
Expression of proteins related to AMPK signaling. **a** Effect of pretreatment with metformin on the AMPK protein in bovine pbMEC treated with LPS. Cells were exposed to LPS for 6 h with or without pretreatment with metformin for 12 h. Afterward, immunofluorescence for p-AMPK (FITC) was performed, and the nuclear was stained with dye DAPI (blue). **b**, **c** Immunoblots and acquisition of intensity from the respective blots. The protein expression was normalized by the respective abundance of GAPDH. Full-length blots are presented in Additional file 1 Fig. S1A. All results are expressed as the means ± SD. Con, control; LPS, lipopolysaccharide; Metf, metformin; ML, LPS with metformin pretreatment. The letters in superscript indicate that the difference between groups was significant (*P* < 0.05)

The expression of proteins related to AMPK signaling is reported in Fig. 2b and c. Phosphorylation of AMPK and ACCα were examined using western blot with phospho-site at T172 for AMPK and at S79 for ACCα. Compared with the control group, metformin increased the ratio of phosphorylated AMPK to total AMPK and the ratio of phosphorylated ACCα to total ACCα (*P* < 0.05). LPS stimulation downregulated phosphorylation of both AMPKα and ACCα as compared with the control group (*P* < 0.05). However, the increased level of phosphorylated AMPKα and ACCα after pretreatment of metformin indicated that this compound reversed the inactivation of AMPK signaling as a result of LPS treatment (*P* < 0.05). Furthermore, we used immunofluorescence in the current study to validate activation of AMPK. As results show in Fig. 2a, metformin significantly enhanced staining of phosphorylated AMPK, while LPS treatment led to a faint level of staining. Thus, LPS challenge did not affect the staining level of AMPK phosphorylation in the metformin-pretreated cells.

### LPS-induced activation of inflammatory genes and proteins is suppressed by metformin pretreatment

**Fig. 3:**
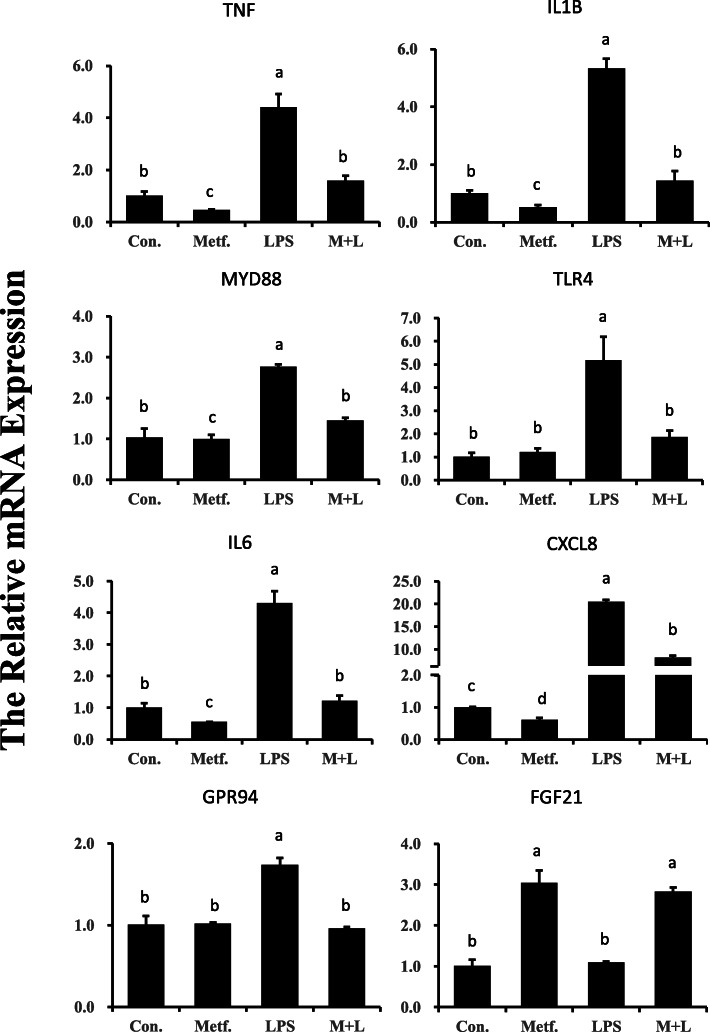
Expression of genes related to inflammation in bovine mammary epithelial cells receiving LPS (2 μg/mL) or pretreatment with metformin (3 mmol/L). Expression of genes was normalized by the geometric mean of the internal control genes (*GAPDH*, *RPS9*, and *UXT*). The expression of genes in Con. group was set as 1.0. The error bars indicate the standard deviation (SD). All results are expressed as the means ± SD. Con, control; LPS, lipopolysaccharide; Metf, metformin; ML, LPS with metformin pretreatment. The letters in superscript indicate that the difference between groups was significant (*P* < 0.05)

The expression of genes related to the inflammatory response is presented in Fig. 3. Compared with the control group, LPS upregulated pro-inflammatory genes including *TNF*, *IL1B*, *IL6* and *CXCL8* (*P* < 0.05). In contrast, compared with control cells, metformin suppressed gene expression of *IL1B, TNFA, IL6* and *CXCL8* (*P* < 0.05). In addition, pretreatment with metformin resisted the upregulation of inflammation in the following LPS challenge. Similarly, LPS stimulation did not affect expression of *TLR4*, *MYD88* and *GPR94* in cells pretreated with metformin. Interestingly, compared with the control group, pretreatment with metformin significantly upregulated *FGF21* gene expression as compared to that in LPS group (*P* < 0.05).

Consistent with expression of genes related to pro-inflammatory factors, compared with the control group, expression of IL-1β and TNF-α proteins were dramatically upregulated in response to LPS stimulation (*P* < 0.05, Fig. 5a, b). However, pretreatment with metformin resisted the upregulated of IL-1b and TNF-a upon stimulation with LPS. Furthermore, the secretion of cytokines (TNF-α and IL-6) in cultured medium increased significantly in the LPS group (*P* < 0.05), while no effect of LPS was observed in cells pretreated with metformin (Fig. 5e).

### NF-κB signaling pathway is inactivated by supply of metformin

**Fig. 4:**
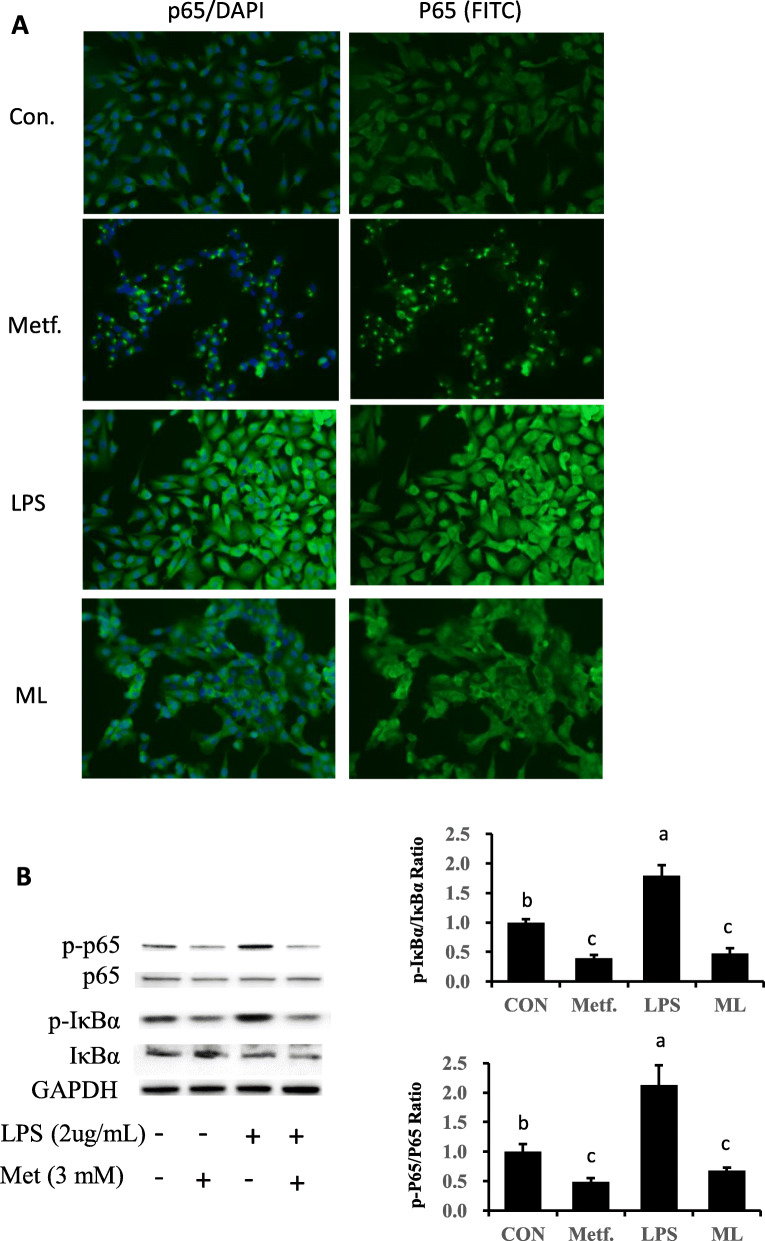
Expression of proteins related to NF-κB signaling. **a** Effect of pretreatment with metformin on the location of NF-κB p65 protein in bovine pbMEC treated with LPS. Cells were exposed to LPS for 6 h with or without pretreatment with metformin for 12 h. Afterward, immunofluorescence for NF-κB p65 (FITC) was performed, and the nuclear was stained with dye DAPI (blue). **b**, **c** Immunoblots and acquisition of intensity from the respective blots. The protein expression was normalized by the respective abundance of GAPDH. Full-length blots are presented in Additional file 1 Fig. S1B. All results are expressed as the means ± SD. Con, control; LPS, lipopolysaccharide; Metf, metformin; ML, LPS with metformin pretreatment. The letters in superscript indicate that the difference between groups was significant (*P* < 0.05)

Activation of NF-κB signaling was assessed by phosphorylation of NF-κB subunit p65 and inhibitor of κBα (IκBα). As data show (Fig. 4b), compared with the control group, phosphorylated p65 exhibited upregulation in LPS-stimulated cells (*P* < 0.05). However, metformin supply reduced the ratio of phosphorylated p65 to total p65 even in after LPS challenge (*P* < 0.05). On the other hand, as inhibitor of κBα, compared to control cells, the ratio of phosphorylated IκBα to total IκBα in LPS stimulated cells was significantly elevated while pretreatment with metformin reversed this effect. In addition to results from western blots analysis, the current study determined the translocation of p65 in bovine mammary epithelial cells using immunofluorescence. As data show (Fig. 4a), compared with control cells, the higher expression and the predominant nuclear location of activated NF-κB (p65) in pbMEC confirmed the inflammatory response after LPS stimulation. However, the LPS-induced activation of NF-κB (p65) was dampened by pretreatment with metformin, leading to lower staining of NF-κB protein in the mammary cell nuclei.

### Inflammasome activation triggered by LPS is dampened by metformin supply

**Fig. 5:**
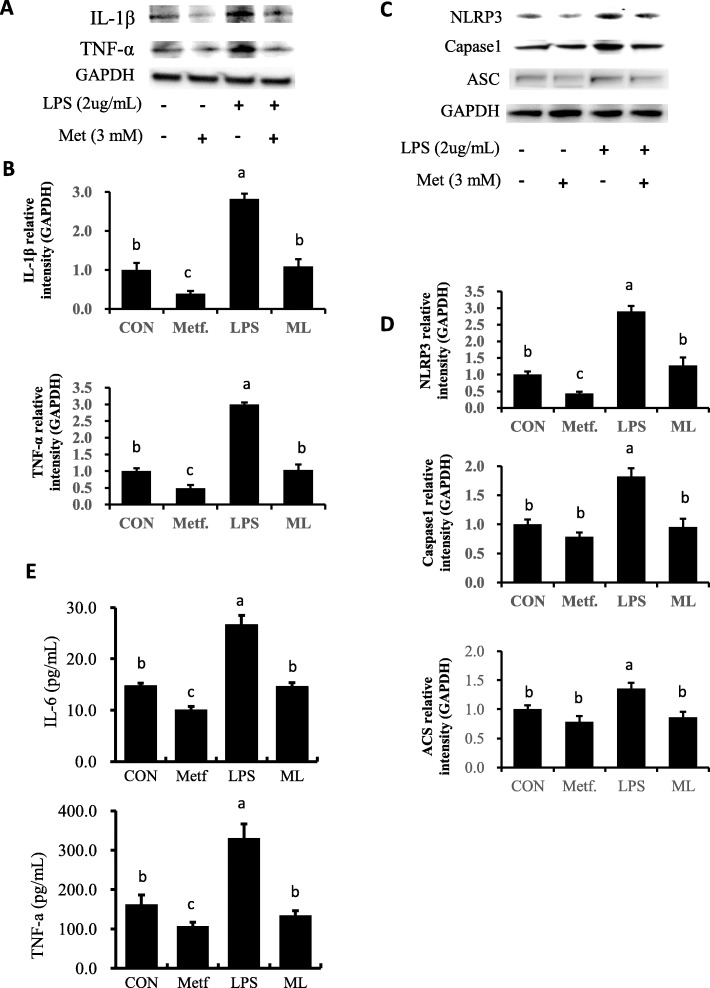
Expression of proteins related to pro-inflammation, inflammasome and cytokines. **a**, **b** Expression of pro-inflammatory proteins. **c**, **d** Expression of proteins related to inflammasome. Immunoblots and acquisition of intensity from the respective blots. The protein expression was normalized by the respective abundance of GAPDH. Full-length blots are presented in Additional file 1 Fig. S1C and S1D. **e** Cytokines secreted into cultured medium by ELISA analysis. All results are expressed as the means ± SD. Con, control; LPS, lipopolysaccharide; Metf, metformin; ML, LPS with metformin pretreatment. The letters in superscript indicate that the difference between groups was significant (*P* < 0.05)

Compared with the control group, LPS challenge remarkably upregulated protein expression of inflammasome components NLRP3, Caspase-1 and ASC (Fig. 5c and d). Additionally, compared with the control group, metformin supply reduced the activation of NLRP3 (*P* < 0.05). As a result, pretreatment of metformin reversed the increase in protein expression triggered by LPS stimulation (*P* < 0.05).

### Metformin regulated H3K14 acetylation level

**Fig. 6:**
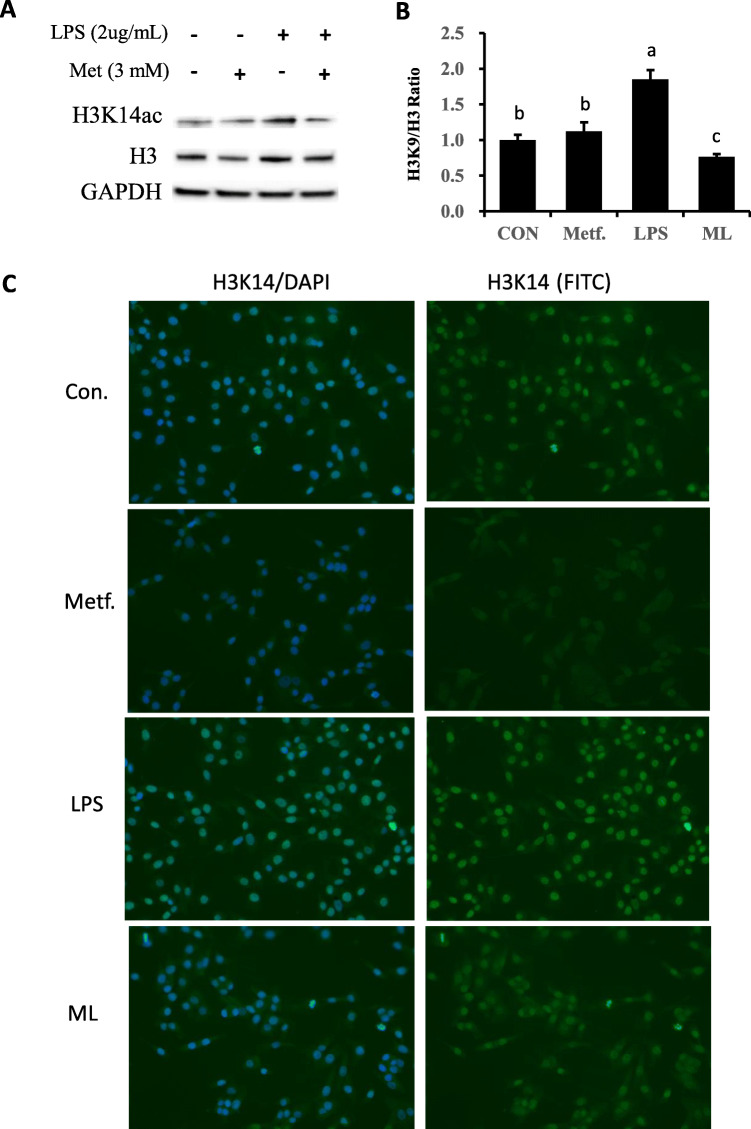
Expression of proteins related to histone H3 and acetylation of H3K14. **a**, **b** Immunoblots and acquisition of intensity from the respective blots. The protein expression was normalized by the respective abundance of GAPDH. Full-length blots are presented in Additional file 1 Fig. S1E. **b**, **c** Effect of pretreatment with metformin on the expression of acetylated histone H3 protein in pbMEC treated with LPS. Cells were exposed to LPS for 6 h with or without pretreatment with metformin for 12 h. Afterward, immunofluorescence for H3K14ac (FITC) was performed, and the nuclear was stained with dye DAPI (blue). All results are expressed as the means ± SD. Con, control; LPS, lipopolysaccharide; Metf, metformin; ML, LPS with metformin pretreatment. The letters in superscript indicate that the difference between groups was significant (*P* < 0.05)

Challenge with LPS induced acetylation of H3 at lysine 14, while this response was reversed by metformin supply prior to LPS challenge (Fig. 6a, b and c). Immunofluorescence confirmed that acetylation of H3 induced by LPS was weakened by addition of metformin.

### Metformin reversed the LPS-induced binding of NF-κB on pro-inflammatory genes promoter

**Fig. 7:**
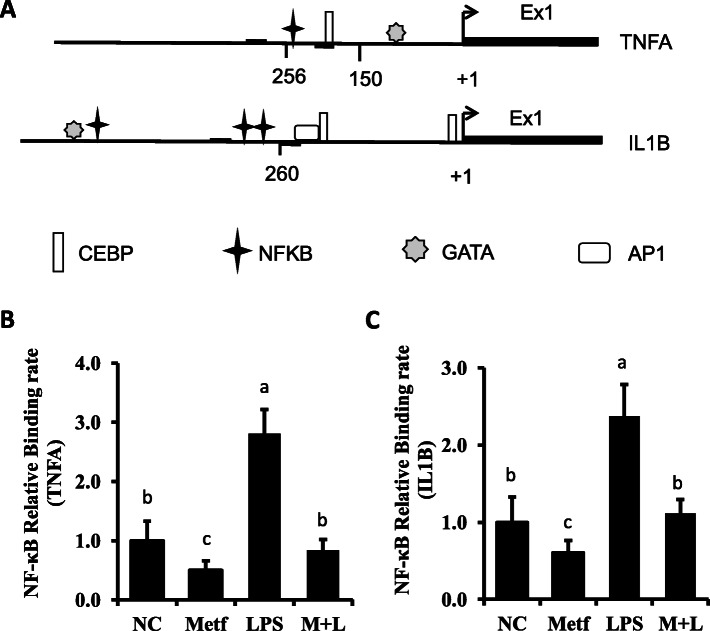
A map for the putative binding sites of transcription factors on the promoter of *TNFA* and *IL1B* and relative binding activity of NF-κB on the promoter of *TNFA* and *IL1B*. **a** Numbers refer to the position relative to the transcriptional starting site, indicated by black arrows. The position of the transcription factors is indicated by the respective symbols. The positions of primers used for chromatin immunoprecipitation assays are denoted by dark lines surround the symbol of NF-κB. The identification for target promoter regions of candidate genes were determined by BLAST analyses as DNA-sequences that are 5′-upstream of the mRNA sequences deposited in the NCBI: NM_173966.3 (TNFA), NM_174093.1 (IL1B). **b** Level of NF-κB binding to the *TNFA* and *IL1B* promoter. Six individual samples in each group were involved in generating chromatin immunoprecipitation analysis. The letters in superscript indicate that the difference between groups was significant (*P* < 0.05)

Putative binding sites for transcription factors were searched for with the PROMO program (http://alggen.lsi.upc.es/cgi-bin/promo_v3/promo/promoinit.cgi?dirDB=TF_8.3). Filters were set ≥0.90 as threshold for similarity of the core sequence (Fig. 7a).

To determine the binding activity influenced by LPS-stimulation and pretreatment with metformin, we examined the immunoprecipitation with specific region of NF-κB putative binding site on both *TNFA* and *IL1B* gene promoter. As data shown in Fig. 7b and c, the average level of NF-κB binding to the *TNFA* and *IL1B* promoter in the LPS groups were increased 2 folds (*P* < 0.05) than that in control cells. However, pretreatment with metformin lowered the binding ability of NF-κB onto both *TNFA* and *IL1B* promoter following the LPS stimulation (*P* < 0.05).

### Pretreatment with metformin reveals effects on cellular energy homeostasis

**Fig. 8:**
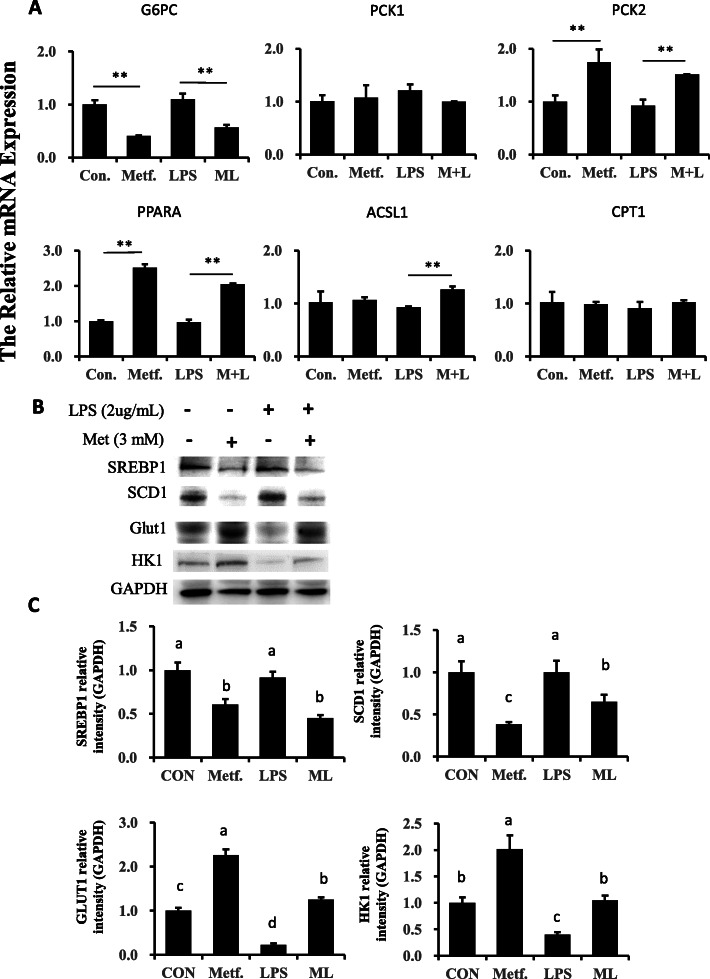
Expression of genes and proteins related to lipid and glucose metabolism. **a** Genes expression of gluconeogenesis and fatty acid oxidation. Expression of genes was normalized by the geometric mean of the internal control genes (*GAPDH*, *RPS9*, and *UXT*). The expression of genes in Con. group was set as 1.0. **b**, **c** Expression of proteins related to lipogenesis and glucose metabolism. The protein expression was normalized by the respective abundance of GAPDH. Full-length blots are presented in Additional file 1 Fig. S1F. The error bars indicate the standard deviation (SD). All results are expressed as the means ± SD. Con, control; LPS, lipopolysaccharide; Metf, metformin; ML, LPS with metformin pretreatment. The letters in superscript indicate that the difference between groups was significant (*P* < 0.05)

Compared with the control group, sterol-regulatory element binding protein 1 (SREBP1) and stearoyl CoA desaturase 1 (SCD1) abundance decreased in the metformin group and LPS-stimulated cells pretreated with metformin (*P* < 0.05, Fig. 8b and c). However, the expression of glucose transporter 1 (GLUT1) in the metformin was upregulated as compared with the control group (*P* < 0.05). The same was true for pretreatment with metformin because after LPS challenge GLUT1 expression was also upregulated compared with cells in the LPS group (*P* < 0.05). Interestingly, compared with the control group, protein expression of hexokinase 1 (HK1) was reduced in LPS-stimulated cells (*P* < 0.05), while metformin pretreatment was effective at reversing this change as indicated by the response in the ML group (*P* < 0.05).

Gene expression of glucose-6-phosphatase (*G6PC*) was lower in the metformin group compared with the control (Fig. 8a), and in cells pretreated with metformin and stimulated with LPS. However, other genes related to gluconeogenesis had different response than *G6PC*. For instance, expression of phosphoenolpyruvate carboxykinase 1 (*PCK1*) was not affected by metformin or LPS treatment. In contrast, compared with control and LPS groups, *PCK2* had greater gene expression in the metformin and ML groups (*P* < 0.05).

In terms of genes related to fatty acid oxidation, compared with the control group, metformin supply led to upregulation of peroxisome proliferator-activated receptor alpha (*PPARA*) mRNA expression. In addition, compared with the LPS group, metformin pretreatment increased the expression of *PPARA* and Acyl-CoA Synthetase Long Chain Family Member 1 (*ACSL1*) (*P* < 0.05). Expression of *CPT1* did not change in the metformin and LPS-stimulated cells.

## Discussion

The key motivation of this study was to determine if activation of AMPK via metformin plays a role in alleviating inflammatory responses and metabolic changes during LPS-stimulation in bovine mammary epithelial cells. If true, the functional and novel role of AMPK together with application of metformin would provide novel strategies besides antibiotics for veterinarians to intervene during cases of acute mastitis. The current study demonstrated a protective effect of metformin on LPS-induced inflammation in mammary cells through specific activation of AMPK signaling and inhibition of NF-κB signaling. In addition, metformin might switch cells from an anabolic to catabolic state to increase energy expenditure during the LPS-induced acute-phase response. Increased histone H3K14 acetylation during LPS stimulation was also reversed by metformin pretreatment, and reduction of histone H3K14 acetylation might account for the decrease in production of cytokines and proteins related to inflammation.

In addition to its main anti-hyperglycemia effect, metformin has a protective effect against oxidative stress and inflammation [32–34]. Several studies have revealed that metformin is a potent activator of the AMPK signaling pathway, which mediates the reduction in the inflammatory response [35–37]. As reported in a recent study, metformin can prevent the development of antibiotic resistance and seems to be promising in terms of its application along with antibiotics during bacterial infections [19]. The elevation of both phosphorylated AMPK and ACCα in response to metformin treatment strongly suggests that metformin plays a pivotal role in the activation of AMPK signaling in pbMEC. We have previously reported that bovine hepatocytes supplied with sodium butyrate remarkably involve activity of AMPK signaling in the regulation of LPS-induced inflammation [12]. Furthermore, there are multiple links between AMPK and immunomodulators in macrophages that have been investigated. Additionally, after pretreatment with metformin in this study, LPS stimulation did not alter activation of AMPK signaling. Together, results indicate that AMPK could be involved in the regulation of LPS-induced inflammatory responses, especially in the regulation of pro-inflammatory factor NF-κB complex.

Metformin has been shown to effectively reduce the expression of inflammation-related molecules, including IL-1β, IL-6 and in the LPS-induced depressive-like behavior in mice [38]. Inflammatory cytokine concentrations were also affected by metformin in a colitis mouse model [39]. Those data were in part replicated in the present study because metformin downregulated expression of *TNF*, *IL1B*, *IL6*, *CXCL8*, *TLR4* and *MYD88* along with downregulation of IL1β and TNFα protein and secretion in LPS-challenged cells. In addition, we also found the anti-inflammatory effect of metformin on LPS-challenged pbMEC with transcriptome data that most inflammatory signaling including cytokines, TNF, NLR and NF-κB signaling were involved in the regulation of metformin supplementation (data not shown). Thus, those data underscored the positive effect of metformin on inflammatory responses in pbMEC.

The transcription factor NF-κB acts as a master switch to regulate a wealth of immune-related genes [40]. Phosphorylated IκBα activates NF-κB and its translocation into nucleus to initiate pro-inflammatory gene transcription [41]. As such, the dampening of phosphorylated IκBα and NF-κB p65 subunit in response to metformin suggested an important immune-modulatory effect. We believe that the suppressed accumulation of nuclear NF-κB by metformin was responsible for the decreased abundance of pro-inflammatory gene transcripts. From a mechanistic standpoint, acetylation of histones could regulate NF-κB-dependent gene accessibility [42]. Although chromatin accessibility for each target pro-inflammatory gene was not determined in this study, the increased deacetylation level of histone H3K14 in cells pretreated with metformin suggested a lack of strength and duration for NF-κB and DNA-binding in the nucleus. Moreover, we found the decreased binding of NF-κB on the promoter of *TNFA* and *IL1B* which indicate the inactivation of NF-κB on the regulation of pro-inflammatory genes transcription by metformin. Alternatively, previous work indicated that activation of the deacetylase SIRT1 induced by AMPK activation could downregulate inflammatory genes via deacetylation of histones [43]. Thus, activated SIRT1 can deacetylate NF-κB and limit the activation of NF-κB and would constitute another mechanism to inhibit inflammatory gene expression. Further studies are required to demonstrate this in bovine mammary cells.

The inhibition of proteins related to the inflammasome including NLRP3, Caspase-1 and ASC in pbMEC after metformin treatment suggested another important biological mechanism of action to control inflammation. Although the mechanism behind the reduction of inflammasome components in pbMEC could not be completely discerned, previous data suggested that metformin inhibits impairment of NLRP3 inflammasome leads to a reduction in IL-1β secretion [44]. Thus, we suspect that NLRP3 activation contributes to maturation and secretion of interleukin 1β and interleukin 6. Furthermore, during cytokine-induced inflammation, ASC is involved in the activation of caspase-1 by mediating the assembly of a caspase-1-inflammasome signaling complex [45]. In agreement, we observed that LPS challenge induced activation of NLRP3, ASC and Caspase1, while metformin pretreatment reversed that effect in the ML group. IT could be possible that the protective effect of metformin on LPS-induced inflammatory response might be mediated through inactivation of NLRP3/Caspase1/ASC inflammasome.

The fact that AMPK is a sensor of cellular energy status led us to suspect that alteration of metabolism in pbMEC during metformin impacts negatively the LPS-induced inflammatory response. The decrease of SREBP1 and SCD1 proteins expression suggested a role of metformin in blocking lipogenesis, a process that requires considerable amounts of ATP [46]. On the other hand, fatty acid oxidation-related genes such as *PPARA* and *ACSL1* were markedly upregulated due to the addition of metformin. Furthermore, upregulation of Glut1 and HK1 protein expression along with the downregulation of *G6PC* reveal the motivation of glycolysis during metformin treatment. Activation of AMPK provides energy to the cell through fatty acid and glucose metabolism in metabolic disorders and inflammatory diseases [10, 11, 36]. In terms of energy expenditure during an immune response, switching from anabolism to catabolism would have been one way whereby metformin modulated the inflammatory response [47]. Notably, activation of AMPK is actually an energy depletion signal to the cells and this may be detrimental to the milk production, such as the inhibitory effect of mTORC1 signaling which is related to casein synthesis [48]. Although the current study mainly uncover the AMPK signaling that contributes to the inflammatory response in bovine mammary cells, further studies would detail the possible negative role of metformin on milk production.

## Conclusion

Taken together, the present research suggested that metformin can activate AMPK signaling and dampen the inflammatory response in bovine mammary epithelial cells experiencing an immune challenge. Suppression of acetylated histone H3 at lysine 14 may lead to inactivation of NF-κB signaling and subsequent downregulation of pro-inflammatory gene expression. Plus, anabolism to catabolism of fatty acid and glucose may result in the increasing capacity of energy provision for anti-inflammation process. Dampened inflammasome signaling may be involved in the regulation of immune response in pbMEC via metformin. Taken together, the results of this study highlight that metformin could play an important role in ameliorating LPS-related induction of pbMEC inflammation via AMPK/NF-κB/NLRP3 signaling.

## Supplementary Information

**Additional file 1 Figure S1.** Uncropped blots images displayed in the context. (Lane 1) Cells untreated with LPS and Metformin. (Lane 2) Cells treated with Metformin (3 mM) and untreated with LPS. (Lane 3) Cells treated with LPS (2 μg/mL) and untreated with metformin. (Lane 4) Cells pretreated with metformin following LPS treatment (2 μg/mL). Red boxes indicated cropped areas presented in main manuscript text.

## Data Availability

The datasets used and/or analyzed during the current study are available from the corresponding author on reasonable request.
