# Supplementary material for: Metformin activated AMPK signaling contributes to the alleviation of LPS-induced inflammatory responses in bovine mammary epithelial cells
Source: BMC Vet Res. 2021 Mar 1;17:97. doi: 10.1186/s12917-021-02797-x (PMC7923493; doi:10.1186/s12917-021-02797-x)

Supplementary files

Metformin activated AMPK signaling contributes to the alleviation of LPS-induced inflammatory responses in bovine mammary epithelial cells

Tianle Xu[1, 2], Xinyue Wu[1], Xubin Lu[1], Yusheng Liang[3], Yongjiang Mao[1], Juan J. Loor[3], Zhangping Yang[1,2,*]

[1] College of Animal Science and Technology, Yangzhou University, Yangzhou, P. R. China 225009

[2] Joint International Research Laboratory of Agriculture and Agri-product Safety of Ministry of Education of China, Yangzhou University, Yangzhou, P. R. China 225009

[3] Mammalian NutriPhysioGenomics, Department of Animal Sciences and Division of Nutritional Sciences, University of Illinois, Urbana 61801

[*] Corresponding author: Zhangping Yang

College of Animal Science and Technology, Yangzhou University, Yangzhou, P. R. China 225009

Tel.: +86-514-87977307, E-mail: yzp@yzu.edu.cn

Additional file 1: Figure S1 Uncropped blots images displayed in the context

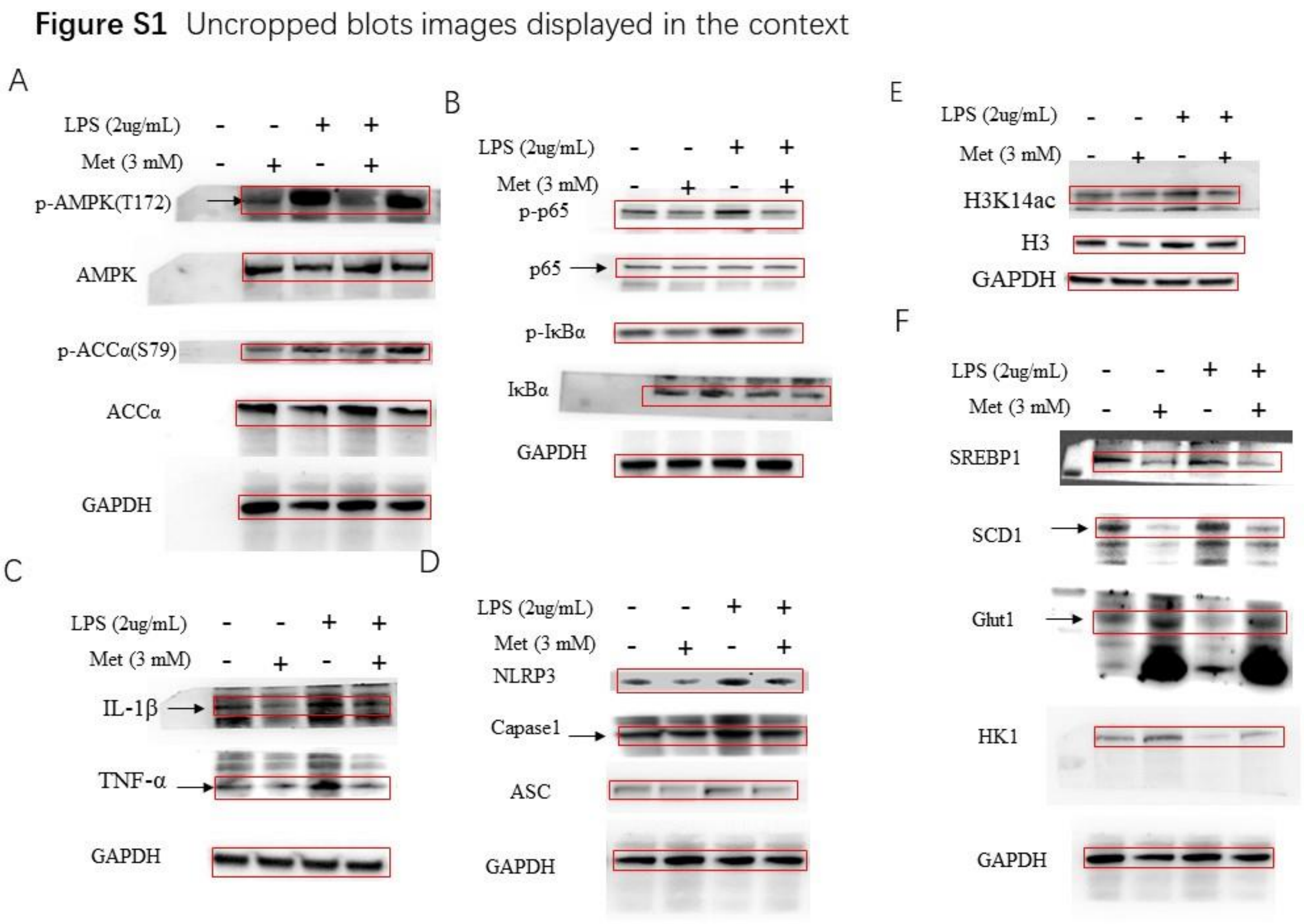

Supplement: Supplementary file 1 — Additional file 1 Figure S1. Uncropped blots images displayed in the context. (Lane 1) Cells untreated with LPS and Metformin. (Lane 2) Cells treated with Metformin (3 mM) and untreated with LPS. (Lane 3) Cells treated with LPS (2 μg/mL) and untreated with metformin. (Lane 4) Cells pretreated with metformin following LPS treatment (2 μg/mL). Red boxes indicated cropped areas presented in main manuscript text. [file 12917_2021_2797_MOESM1_ESM.pdf]
